# Static Solid Relaxation Ordered Spectroscopy: SS-ROSY

**DOI:** 10.3390/ijms20235888

**Published:** 2019-11-24

**Authors:** Gregory S. Boutis, Ravinath Kausik

**Affiliations:** 1 Department of Physics, Brooklyn College of the City University of New York, 2900 Bedford Avenue, Brooklyn, NY 11210, USA; 2 Department of Physics, The Graduate Center of the City University of New York, 365 5th Ave, New York, NY 10016, USA; 3 Schlumberger-Doll Research, 1 Hampshire Street, Cambridge, MA 02139, USA; RViswanathan@slb.com

**Keywords:** inverse Laplace transform, multiple pulse NMR, correlation spectroscopy

## Abstract

A two-dimensional pulse sequence is introduced for correlating nuclear magnetic resonance anisotropic chemical shifts to a relaxation time (e.g., T_1_) in solids under static conditions. The sequence begins with a preparatory stage for measuring relaxation times, and is followed by a multiple pulse sequence for homonuclear dipolar decoupling. Data analysis involves the use of Fourier transform, followed by a one-dimensional inverse Laplace transform for each frequency index. Experimental results acquired on solid samples demonstrate the general approach, and additional variations involving heteronuclear decoupling and magic angle spinning are discussed.

## 1. Introduction

One (1D) and two dimensional (2D) inverse Laplace transforms (ILTs) have facilitated the characterization of exponential decays in a wide range of diffusion or relaxation nuclear magnetic resonance (NMR) experiments [1,2]. A number of different experiments have been reported in the literature that make use of the ILT including exchange [3], relaxation–relaxation [1,4], relaxation–diffusion [5], diffusion–diffusion [6], and chemical shift–diffusion [7] correlation approaches. These methods have been successfully applied to probe molecular dynamics and structure, for example via a diffusion coefficient or exchange rate, in many different type of systems such as polymers [8,9], rock [10,11], and biological systems [12,13,14].

The one field of applications where such approaches may still be considered nascent are for the characterization of constituents and phases in amorphous and disordered solids. Current approaches to obtaining high resolution chemical shift information of solids in high field laboratory systems often make use of fast magic-angle spinning, hetero- and/or homo-nuclear multiple pulse methods or combinations of these approaches, by selectively averaging a systems’ nuclear spin Hamiltonian(s) in physical and/or spin space [15]. In portable NMR systems, or in samples where magic-angle spinning cannot be used, alternative approaches for resolving chemical shifts and relaxation times are often needed. One approach, recently introduced by Lupulescu and coworkers, is to distinguish nuclear spins in a solid when they have markedly different relaxation times [16]. The method introduced in that work made use of non-negative least-squares fitting of relaxation data to obtain a 2D mapping of relaxation time (e.g., $T_{1}$) to chemical shift.

In this work we introduce a new methodology for correlating anisotropic chemical shifts to relaxation times in a 2D experiment of static solids. Homonuclear dipolar decoupling based on multiple pulse methods is introduced together with Laplace inversion of the relaxation data. This 2D pulse sequence is termed static solid relaxation ordered spectroscopy, SS-ROSY. A homonuclear decoupled spectrum is acquired as a function of a relaxation delay (e.g., in a saturation recovery experiment). A one-dimensional ILT [1,2] is performed for every frequency index, following Fourier transformation, resulting in a mapping that correlates anisotropic chemical shift to relaxation time(s). The acronym ROSY (Relaxation Ordered SpectroscopY) has appeared previously in the literature [17], and seminal work on ROSY has also been demonstrated in NMR [18], and electron paramagnetic resonance [19]. However, this work highlights the use of homonuclear decoupling for static solid samples in tandem with the ILT methods. The inverse Laplace method used here has been introduced on some commercial NMR spectrometers and makes the approach for measuring relaxation times both feasible and user-friendly in comparison to non-negative least-squares fitting approaches. Additionally, this approach allows for a user friendly method of studying the distribution of relaxation times in a heterogenous samples, as ILT algorithms have become commercially available. We highlight experimental data on a single crystal of calcium fluoride (CaF${}_{2}$) whose ${}^{19}$F natural line-width is reduced by homonuclear dipolar decoupling; the ${}^{19}$F spin lattice relaxation time ($T_{1}$) of the nuclei of the crystal is correlated to their anisotropic chemical shift in a 2D mapping following signal processing. Additional variations of the approach are discussed making use of homonuclear decoupling combined with magic angle spinning approaches.

## 2. Results

**Figure 1:**
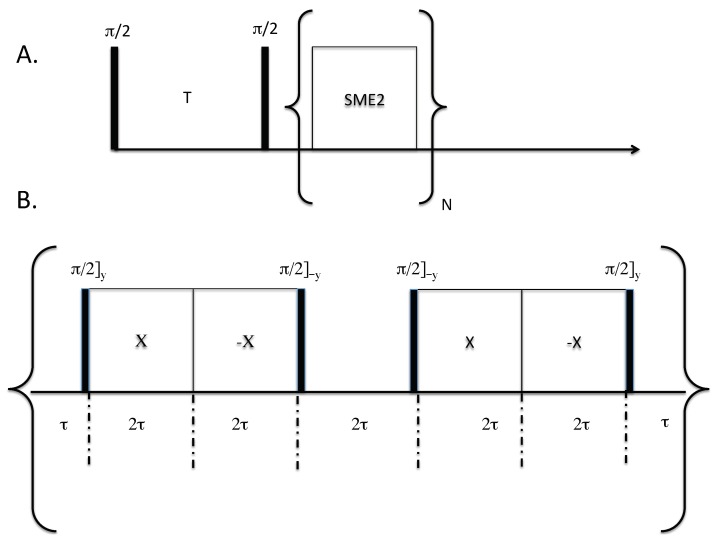
(**A**) Two-dimensional anisotropic chemical shift-relaxation correlation experiment. An initial stage, involving a saturation recovery, followed by a stroboscopically-detected symmetric magic echo train (SME2) dipolar decoupling sequence [20]. The following two step phase cycling scheme was implemented: Only the initial excitation pulses (in practice many π/2 pulses are used to saturation the magnetization, not just one as shown in our schematic) was varied (*x,-x*) and the receiver phase was varied as (*y,-y*) per scan. (**B**) The SME2 sequence for homonuclear dipolar decoupling [20]. For stroboscopic detection, only one point was collected at the end of an SME2 cycle. The acquisition time must be included in the delays, so as to maintain a total cycle time of 12τ. Acquisition occurs at the end of the SME2 cycle, in the middle of the delay 2τ.

The relaxation–chemical shift dispersion experiment is shown in Figure 1. In this sequence, nuclear spins are first saturated with a π/2 pulse. Figure 1 only shows one saturation pulse for simplicity, but in practice many π/2 pulses are applied to saturate the magnetization. Magnetization is recovered as a function of the variable delay T and subsequently read out using a symmetric magic echo train (SME2) sequence [20]. The SME2 sequence is comprised of a train of symmetric magic echo trains that refocus the evolution of the ${}^{19}$F–${}^{19}$F homonuclear dipolar interactions; magnetization is stroboscopically detected at the cycle time of 12τ. Under this sequence, chemical shift anisotropy, resonance offset, and static field inhomogeneity are not removed while homonuclear dipolar interactions are. In principle, any multiple pulse sequence may be used in place of the SME2 sequence that coherently averages the homonuclear dipolar interaction (e.g., the Mansfield-Rhim-Elleman-Vaughn-8 sequence [21]).

Two dimensional relaxation–chemical shift correlation data may be described by the expression [16]

(1) $$S\left(T,t\right)=\int \int \;I\left(\nu ,R\right)e^{i\nu t}G\left(R,T\right)\;d\nu \;dR$$

where I(ν,R) represents the line-shapes of different sites within the sample with characteristic relaxation rates *R*. The function G(R,T) represents the kernel for the relaxation process (e.g., in a saturation recovery experiment $G\left(R,T\right)=1-exp\left[-TR_{1}\right]$). Obtaining I(ν,R) from S(T,t) involves a Fourier transform of S with respect to the time *t*, which is stroboscopically measured by multiple pulse decoupling (for the SME2 sequence used in this work *t* = 12τ). A 2D mapping of frequency ν (or chemical shift) to relaxation time is obtained by performing an inverse Laplace transform with respect to the variable T. Conditions for the stability and reliability of the one dimensional inverse Laplace transform used in this study depend largely on signal to noise and the sampling of times chosen in the T domain (see Figure 1A). The details for the Butler–Reeds–Dawson algorithm used in this study have been discussed in prior work, which include simulations of the performance of the algorithm with low signal to noise data and the sensitivity of the output of the algorithm with the regularization parameter α (see [1,2] ). We found that the algorithm we implemented produced results (the peak relaxation time value in the relaxation dimension) similar to what was obtained by least squares fitting, however, was sensitive to the number of samples collected in the relaxation dimension as well as signal to noise. We did not fully characterize how these experimental parameters varied in cases of overlapped spectra, or in the different samples studied.

**Figure 2:**
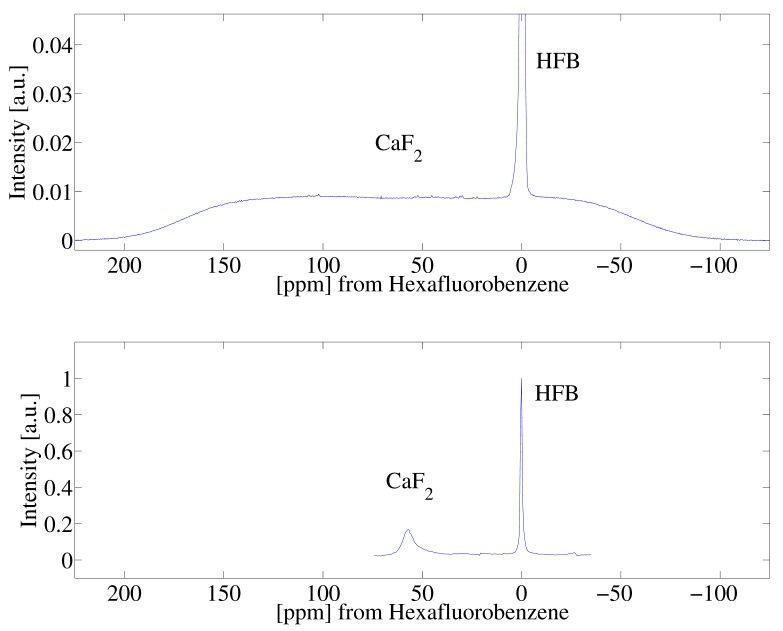
(**A**) Single-pulse ${}^{19}$F nuclear magnetic resonance (NMR) spectra of a CaF${}_{2}$ crystal and HFB. As described in the experimental section, the two samples were simultaneously in the radiofrequency coil, held in separate capillary tubes. (**B**) ${}^{19}$F dipolar decoupled NMR spectrum of CaF${}_{2}$ and HFB following a π/2 pulse and stroboscopically detected acquisition of the SME2 sequence.

Figure 2A highlights experimental data of the spectrum of the single crystal CaF${}_{2}$ and HFB sample following a single π/2 pulse. We note that the two spin systems were isolated from one another in the experiment, held in separate capillary tubes while the experiment was performed. The line-width of the calcium fluoride signal is broad and featureless owing to the strong ${}^{19}$F homonuclear dipolar interactions. Superimposed on the spectrum is the narrow signal arising from the HFB liquid. Figure 2B shows experimental results of the spectra acquired by the SME2 sequence measured stroboscopically following a single π/2 pulse. The spectral bandwidth of the single pulse experiment shown in Figure 2A, and the stroboscopic experiment in Figure 2B are different and are not equal owing to the nature of the stroboscopic experiment (spectral bandwidth = 1/dwell time). The SME2 sequence achieves excellent line-narrowing ability reducing the natural line-width of CaF${}_{2}$ from approximately 200 ppm to 5 ppm, allowing for a measure of the anisotropic chemical shift of ${}^{19}$F spins in the crystal (the ${}^{19}$F spins of HFB were referenced to 0 ppm).

**Figure 3:**
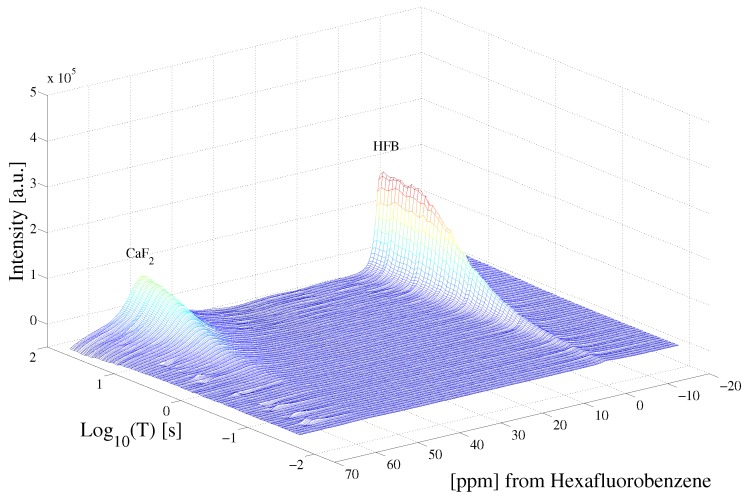
Saturation recovery of the ${}^{19}$F dipolar decoupled CaF${}_{2}$ and HFB NMR spectra, following the pulse sequence shown in Figure 1. Each spectrum was acquired at variable delay T, shown in Figure 1.

**Figure 4:**
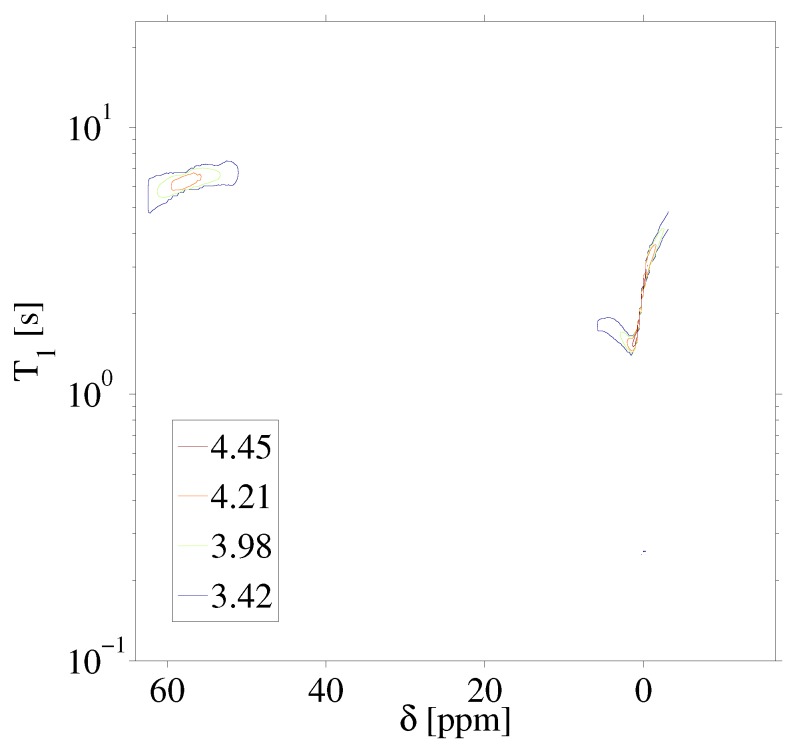
Inverse Laplace transform of the 2D ${}^{19}$F anisotropic chemical shift-relaxation correlation data shown in Figure 3. Details of the inverse Laplace conditions are provided in the text. Contour line intensities, in color, are shown on a logarithmic scale.

Figure 3 highlights the raw data of the saturation recovery of the CaF${}_{2}$ and HFB for the 70 experiments performed. The ${}^{19}$F spins of CaF${}_{2}$, shifted at 57 ppm relative to HFB, exhibit a slightly longer T${}_{1}$ than the ${}^{19}$F of HFB. A 1D ILT, described in [2], was applied for each frequency index in the data. The algorithm made use of the Butler–Reeds–Dawson method [22], with α converging typically in the range of approximately 10${}^{-3}$. The data shown in Figure 4 highlight the result of the ILT, and correlate the anisotropic chemical shift to the T${}_{1}$ for the ${}^{19}$F nuclei in CaF${}_{2}$ and in HFB. Prior approaches for resolving the relaxation domain in a 2D chemical shift-relaxation experiment, without homonuclear decoupling, have made use of non-negative least-squares fitting [16], or non-negative Tikhonov fitting [23]. The ILT used in this work is commercially available and integrated on some NMR systems, including portable spectrometers, making the approach easy to apply in a wide range of solids applications. We note a dispersion of the T${}_{1}$ relaxation time for HFB, which is unexpected for a liquid. This dispersion was found to be sensitive to the number of points included in the search space in the relaxation dimension, and we suspect may be made smaller by including more experimental points in the indirect dimension. We point out that the use of ILT methods to study the $T_{1}$ of ${}^{19}$F spins in HFB has not been investigated prior to this work, to our knowledge. It has been documented that HFB, which forms molecular complexes, undergoes anisotropic motion at 25 °C [24] with two different correlation times, which may also contribute to distributed $T_{1}$ values observed in Figure 4.

**Figure 5:**
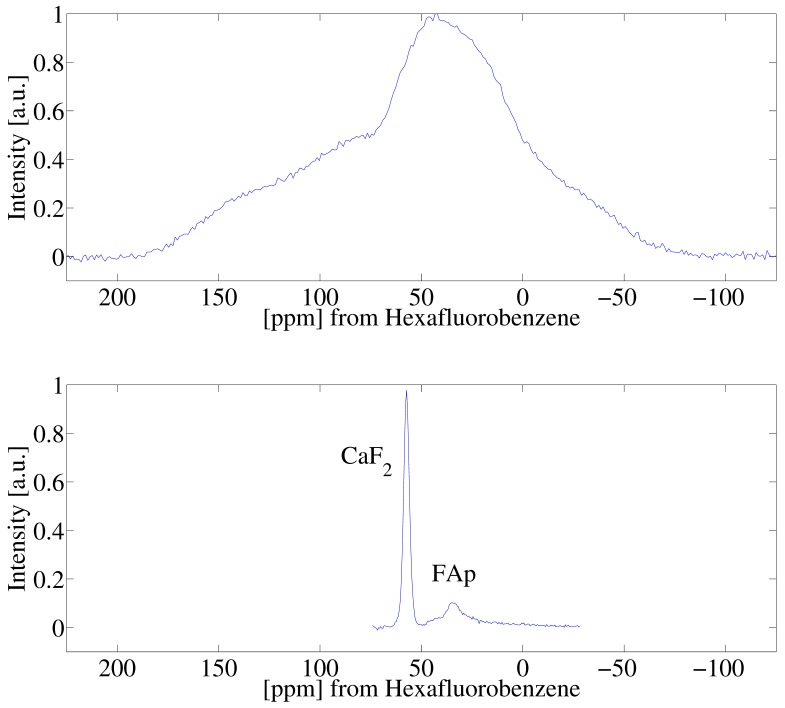
(**A**) Single pulse ${}^{19}$F NMR spectra of a CaF${}_{2}$ and FAp crystal. (**B**) ${}^{19}$F dipolar decoupled NMR spectrum of CaF${}_{2}$ and FAp following a π/2 pulse and stroboscopically detected acquisition of the SME2 sequence. We note the signal intensities for the two samples are different owing to the different crystal sizes, and the respective concentration of fluorine nuclei.

**Figure 6:**
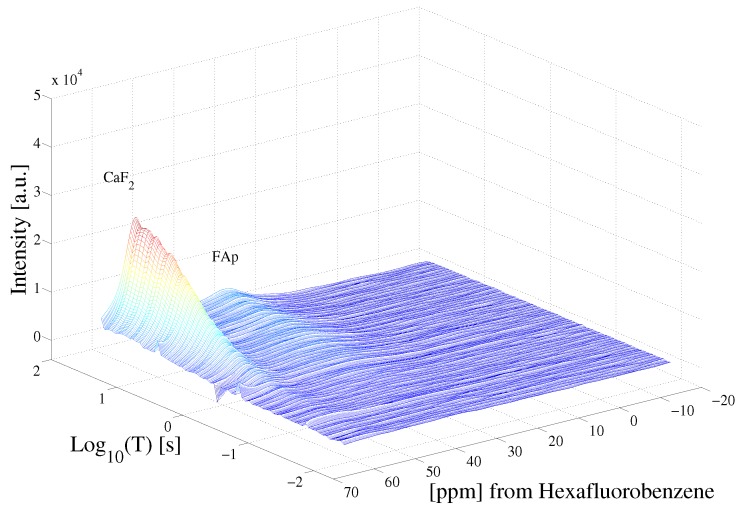
Saturation recovery of the ${}^{19}$F dipolar decoupled CaF${}_{2}$ and FAp NMR spectra, following the pulse sequence shown in Figure 1. Each spectrum was acquired at variable delay T, shown in Figure 1.

**Figure 7:**
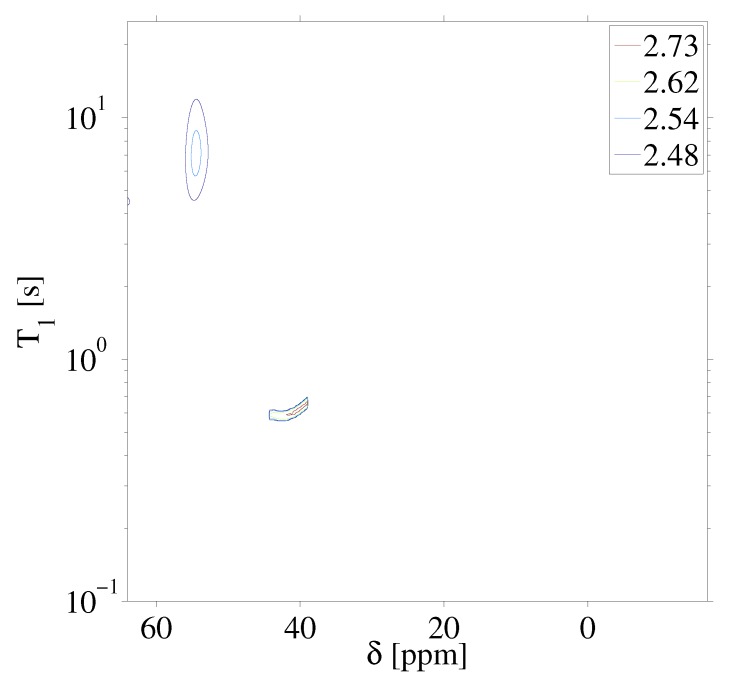
Inverse Laplace transform of the 2D ${}^{19}$F anisotropic chemical shift-relaxation correlation data shown in Figure 6. Details of the inverse Laplace conditions are provided in the text. Contour line intensities, in color, are shown on a logarithmic scale. We note that the CaF${}_{2}$$T_{1}$ for these measurements is different than that shown in Figure 4 as a different crystal and different orientation was used.

To further demonstrate the procedure on a system of two solids, experiments were performed on a single crystal of CaF${}_{2}$ and a sample of fluoroapatite (FAp). Figure 5 highlights the experimental results following (A) a single π/2 pulse and (B) the chemical shift resolved spectrum resulting from the the application of the stroboscopically detected SME2 sequence shown in Figure 1. The ${}^{19}$F nuclear spins in CaF${}_{2}$ and FAp have markedly different chemical shifts owing to the crystalline structure of the samples. Figure 6 highlights the raw saturation recovery data for this sample consisting of two crystals, and in Figure 7 we show the resulting inverse Laplace data highlighting the correlation of relaxation time to anisotropic chemical shift. We note that the $T_{1}$ shown in Figure 7 for CaF${}_{2}$ is slightly different than that shown in Figure 4, as a different crystal was used at a different orientation. In principle, the experimental approach described here may be used to study the angular dependence of $T_{1}$ anisotropy time in solids by rotating the solid. We note that the chemical shift of single crystal CaF${}_{2}$ does not have strong angular dependence, but the spin lattice relaxation varies with the crystalline orientation with respect to the Zeeman field [25]. The $T_{1}$ relaxation time in single crystal CaF${}_{2}$ depends on the Zeeman spin diffusion rate, impurity concentration, the correlation times of impurities, as well as the Zeeman field strength [26].

## 3. Discussion

In some systems where chemical shifts of various nuclear spins overlap but have large differences in relaxation times, the addition of the relaxation dimension may allow for visualizing the various anisotropic chemical shifts more so than that achieved in a 1D decoupled experiment. This approach was demonstrated in [16], however, without homonuclear decoupling. Additional variations of the approach shown schematically in Figure 1A are possible for studies of solids without magic angle spinning. Heteronuclear decoupling may be applied on I spins, whilst detecting the relaxation and anisotropic chemical shift information for S spins, in a two spin IS system (e.g., ${}^{13}$C–${}^{1}$H spin systems). In this case, the SME2 sequence would be replaced with heteronuclear decoupling on the I spins and detecting on the S spins. Or, experiments may be performed in tandem with these methods with fast magic-angle spinning which further average dipolar interactions and allow for resolving isotropic chemical shifts. Lastly, other relaxation times may be readily probed; the saturation recovery may be replaced with a spin-locking pulse to measure a rotating frame relaxation time $T_{1\rho }$, a train of π pulses to measure a spin-spin relaxation time T${}_{2}$, or a π pulse may be used if an inversion recovery is preferred.

The application of these methods especially on low field and mobile NMR systems under static conditions could have important applications on different industries, including oil and gas exploration. This is particularly true for the characterization of shale rock which has established itself as a premier energy resource in recent years [27]. Shale rock resources include tight oil shale and gas shale plays and are characterized by the presence of organic carbon in different phases. These include the solid organic carbon known as kerogen, which is defined as the phase that is insoluble in organic solvents, the highly viscous bitumen, which is soluble in organic solvents, the lighter hydrocarbon oil and gas. The ability to characterize these different components in the lab or at the well-site through the application of quick NMR methods would enable the possibility of improving the exploration and field development plans. While multi-dimensional relaxation–diffusion experiments have already found to be useful for the characterization of shale rocks [28,29,30] the challenge for characterizing the solid and viscous components still exists. Recently the application of solid state NMR techniques has proved to be promising for the understanding of these components [31,32,33]. Two-dimensional relaxation–chemical shift measurements on static solids as introduced in this paper can potentially help extend this understanding and also aid in well-site cuttings analysis providing near real-time information.

## 4. Materials and Methods

All NMR experiments were performed at a ${}^{19}$F Larmor resonance frequency of 168.84 MHz, making use of a homebuilt probe capable of delivering short radiofrequency pulses. The radiofrequency coil was approximately 0.8 cm long and 0.2 cm in diameter, and consisted of seven turns of flat copper wire. All π/2 pulses were 2.0 μs in duration. For the SME2 sequence (refer to Figure 1B), the τ spacing was set to 5 μs. In all stroboscopic experiments, only one point was acquired per cycle. The dwell time in stroboscopic experiments was set equal to 12τ which includes the π/2 pulse widths and the acquisition time of one data point. In the second dimension, the SME2 cycle was applied 512 times, and 70 experiments in the T${}_{1}$ domain were acquired ranging from 1 ms to 50 s, spaced equally on a logarithmic scale. One sample used in the experiments was a single crystal of CaF${}_{2}$ sealed in one capillary tube, and a second capillary tube containing hexafluorobenzene (HFB). A second sample was comprised of a single crystal of CaF${}_{2}$ and a single crystal of fluorapatite (FAp). Both liquid and crystalline samples were placed in a capillary tube having an inner diameter of 1.5 mm. All experiments were performed at approximately 22 °C, under static conditions (without magic-angle spinning).

## 5. Conclusions

An approach for performing 2D anisotropic chemical shift-relaxation correlation experiments in static solids is introduced. Homonuclear dipolar-decoupling is applied to a spin system as a function of a preliminary stage that probes a relaxation time (e.g., by saturation recovery). The generalized approach makes use of Fourier transform of the data, followed by a 1D ILT for every frequency index. Experimental results are shown on a single crystal of CaF${}_{2}$ and FAp that made use of a multiple pulse sequence designed to selectively average the ${}^{19}$F homonuclear dipolar interactions. Additional variations of the experiment are discussed, including making use of homonuclear decoupling approaches in tandem with magic-angle spinning.

## Abbreviations

The following abbreviations are used in this manuscript:

ILT	Inverse Laplace Transform
NMR	Nuclear Magnetic Resonance
RF	Radio Frequency
MREV-8	Mansfield-Rhim-Elleman-Vaughn-8 sequence (a multiple pulse decoupling cycle)
SME2	Symmetric Magic Echo Train-2 sequence (a multiple pulse decoupling cycle)
HFB	Hexafluorobenzene
CaF${}_{2}$	Calcium Fluoride
FAp	Fluorapatite
